# Chemical and Transcriptomic Analyses Provide New Insights into Key Genes for Ginsenoside Biosynthesis in the Rhizome of *Panax japonicus* C. A. Meyer

**DOI:** 10.3390/molecules29204936

**Published:** 2024-10-18

**Authors:** Qichun Yang, Chao Xiong, Jiao Zhang, Yue Ming, Shaopeng Zhang, Limei Wang, Hongxun Wang, Ran Xu, Bo Wang

**Affiliations:** 1 School of Life Science and Technology, Wuhan Polytechnic University, Wuhan 430023, China; yangqc0201@163.com (Q.Y.); xiongchao080190@126.com (C.X.); m15972485912@163.com (Y.M.); shaopeng@whpu.edu.cn (S.Z.); wanglimeiyx@163.com (L.W.); wanghongxunhust@163.com (H.W.); 2 State Key Laboratory Breeding Base of Dao-di Herbs, National Resource Center for Chinese Materia Medica, China Academy of Chinese Medical Sciences, Beijing 100700, China; zhangjiao2107@163.com; 3 Hubei Institute for Drug Control, Hubei Provincial Drug Quality Inspection and Control Technology Research Center, Wuhan 430075, China

**Keywords:** *Panax japonicus*, transcriptome, triterpenoid saponin, UGTs

## Abstract

*Panax japonicus* C. A. Meyer is renowned for its significant therapeutic effects and is commonly used worldwide. Its active ingredients, triterpenoid saponins, show variation in content among different tissues. The tissue-specific distribution of saponins is potentially related to the expression of vital genes in the biosynthesis pathway. In this study, the contents of five saponins (ginsenoside Ro, chikusetsusaponin IV, chikusetsusaponin IVa, ginsenoside Rg1, and ginsenoside Rb1) in three different tissues were determined by HPLC. Transcriptome sequencing analysis identified differentially expressed genes (DEGs) involved in triterpenoid saponin biosynthesis, highlighting significant correlations between saponin contents and the expression levels of 10 cytochrome p450 monooxygenase (CYP) and 3 UDP-glycosyltransferase (UGT) genes. Cloning, sequencing, and prokaryotic expression of UGT genes confirmed the molecular weights of UGT proteins. Gene sequence alignment and phylogenetic analysis provided preliminary insights into UGT gene functions. Meanwhile, the function of one UGT gene was characterized in the yeast. These findings advance our understanding of the triterpenoid saponin biosynthesis in *P. japonicus* and support future research in traditional Chinese medicine (TCM) and synthetic biology.

## 1. Introduction

*Panax japonicus* C. A. Meyer, a member of the *Araliaceae* family and *Panax* genus, plays a significant role in traditional Asian medicine. Its rhizome extracts have a variety of pharmacological characteristics, such as reducing blood lipids [1] and having anti-cancer [2] and anti-inflammation effects [3]. Furthermore, *P. japonicus* contains compounds similar to those in *P. ginseng* and *P. notoginseng*; it is often employed as an alternative to *P. ginseng* in practical applications [4]. Like other *Panax* genus medicinal plants, the main active ingredient of *P. japonicus* is triterpenoid saponin [5], predominantly oleanane-types (e.g., ginsenoside Ro, chikusetsusaponin IV, and IVa) with a minority of dammarane-types (e.g., ginsenoside Rg1 and Rb1). In *Panax* species, the distribution and content of saponins vary across different tissues. For example, the distribution patterns and contents of different types of saponins differ among the leaves, stems, rhizomes, and lateral roots of *P. japonicus* [6]. Additionally, UHPLC-QTOF-MS analysis reveals differential saponin contents among the cork, cortex, and phloem of *P. ginseng* [7]. These results indicate that different types of saponins appear to accumulate in a tissue-specific manner. Therefore, the rhizome of *P. japonicus* was divided into three types of tissues (periderm, cortex, and stele) to investigate their differences in saponin contents, thereby facilitating the cost-effective utilization of *P. japonicus* resources.

There exists a relationship between the distribution and accumulation of natural products and the degrees of expression of related enzymes in plant tissues [8]. Saponins from *P. japonicus*, primarily ginsenosides and chikusetsu saponins, are synthesized in two main steps: triterpenoid skeleton synthesis and post-synthesis modification of the skeleton [9]. The mevalonate pathway (MVA) and methylerythritol phosphate pathway (MEP) are the sources of triterpenoid skeleton production and primarily produce the precursor isopentenyl pyrophosphate (IPP) through the MVA pathway [10]. IPP is transformed into 2,3-oxidosqualene by a series of enzymes, including farnesyl diphosphate synthase (FPS) and squalene synthase (SS). Subsequently, oxidosqualene cyclases (OSCs) catalyze the formation of various triterpenoid skeletons and sterols from 2,3-oxidosqualene [11]. Various enzymes, including beta-amyrin synthase (β-AS), cytochrome P450 monooxygenase (CYP), and glycosyltransferase (GT), transform the triterpenoid skeleton into diverse triterpenes with distinct species and functionalities via chemical modifications such as oxidation, substitution, and glycosylation [12]. At present, research remains limited on how gene expression influences saponin distribution and accumulation across the different tissues of *P. japonicus* rhizomes.

In this work, we aim to elucidate the transcriptome and metabolome of the periderm, cortex, and stele of the *P. japonicus* rhizome. The alterations in triterpenoid saponin in *P. japonicus* were examined with high-performance liquid chromatography (HPLC). Using the Illumina HiSeq platform, we obtained transcriptome data from three tissues and compared their gene expressions. We also identified key enzyme genes associated with saponin biosynthesis and correlated saponin distribution with tissue-specific gene expression. Additionally, UGT genes from *P. japonicus* were cloned and expressed in order to characterize the catalytic function. These results offer a foundation for additional studies on the saponin biosynthesis in *P. japonicus* as well as a guide for the future exploration and utilization of the rhizome resources of *P. japonicus*.

## 2. Results

### 2.1. Saponin Contents of P. japonicus

**Figure 1:**
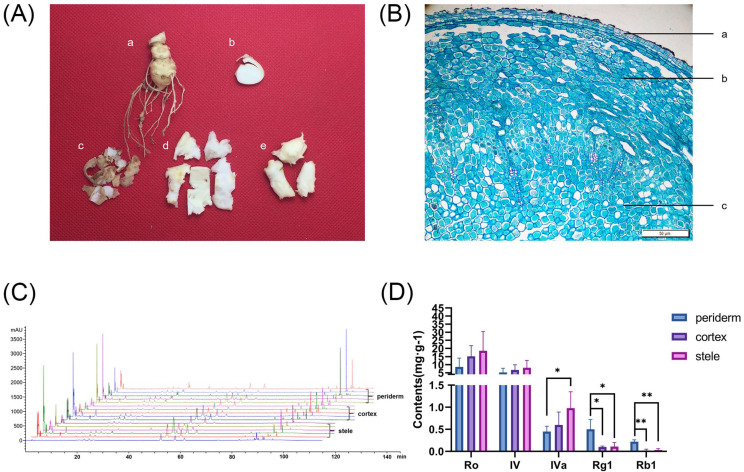
HPLC chromatograms and saponin contents in three rhizome tissues of *P. japonicus*. (**A**) Photograph of different tissues of *P. japonicus* rhizome: (a) rhizome, (b) cross section of rhizome, (c) periderm, (d) cortex, and (e) stele. (**B**) Cross-sectional microscopic structure of the *P. japonicus* rhizome: (a) periderm, (b) cortex, and (c) stele. (**C**) HPLC chromatogram profiles. (**D**) Contents of the five saponins in periderm, cortex, and stele. * *p* < 0.05, ** *p* < 0.01.

During the HPLC analysis, we determined the contents of ginsenoside Ro, chikusetsusaponin IV, chikusetsusaponin IVa, ginsenoside Rg1, and ginsenoside Rb1 in periderm, cortex, and stele, respectively (Figure 1). The total content of five saponins in the periderm, cortex, and stele was 15.02 mg/g, 22.66 mg/g, and 27.71 mg/g, respectively. And the content of oleanane-type ginsenosides, i.e., ginsenoside Ro, chikusetsusaponin IV, and chikusetsusaponin IVa, in these three tissues was 14.3 mg/g, 22.54 mg/g, and 27.71 mg/g, respectively. In contrast, the content of dammarane-type ginsenosides, i.e., ginsenoside Rg1 and ginsenoside Rb1, was as low as 0.72 mg/g, 0.12 mg/g, and 0.14 mg/g in the periderm, cortex, and stele, respectively. Among the five saponins, ginsenoside Ro had the highest content in all three tissues, while ginsenoside Rb1 had the lowest content in all three tissues. These findings suggested that various rhizome tissues have divergent propensities for the accumulation of distinct saponin types and that the predominant accumulation of saponins in the stele may be associated with its main functions of nutrient storage and nutrient transport within the plant.

### 2.2. Transcriptomic Analysis in P. japonicus

The transcriptomes of the periderm, cortex, and stele were analyzed using Illumina HiSeq sequencing technology to provide more information on the saponin biosynthesis mechanism in *P. japonicus*. In total, 55 Gb of data were acquired, with each sample yielding an average output of 6.11 Gb. The 367 M clean reads, averaging 150 bp in size, were employed for the de novo assembly after filtering. Ultimately, 777,642 contigs averaging 754 bp in size were generated, resulting in the assembly of 119,882 unigenes. With an average contig size of 1052 bp and an N50 contig size of 1691 bp, the GC content stood at 41.3% (Table S3).

### 2.3. Functional Annotation of the Transcriptome

In total, 85,678 unigenes yielded significant BLAST results against public databases, and 31,554 unigenes (36.8%) were matched across all four databases. Among them, roughly 10,016 (11.7%) of the matches with the Nr database were significant. Furthermore, significant matches were found in the KEGG, COG, and Swiss-Prot databases for 838, 126, and 531 unigenes, respectively (Figure S1).

The COG database search showed that 26 function categories housed 58,581 unigenes (Figure S2). The primary COG category was “general functional prediction only” (10,243), followed by “transcription” (5482), “replication, recombination, and repair” (4790), and “posttranslational modification, protein turnover, chaperones” (4673). Additionally, 1448 uni-transcripts belonged to the category “secondary metabolite biosynthesis, transport, and catabolism”and may play critical roles in the regulation of secondary metabolite biosynthesis.

Moreover, a total of 295,522 sequences were classified into three primary GO categories (Figure S3). These GO terms were subdivided into 56 subcategories. Among the biological process category, cellular, metabolic, and single-organism processes were the most prominently represented terms. Notably, 29,305 sequences belonged to a metabolic process category, indicating their potential involvement in the metabolite biosynthesis pathway.

**Figure 2:**
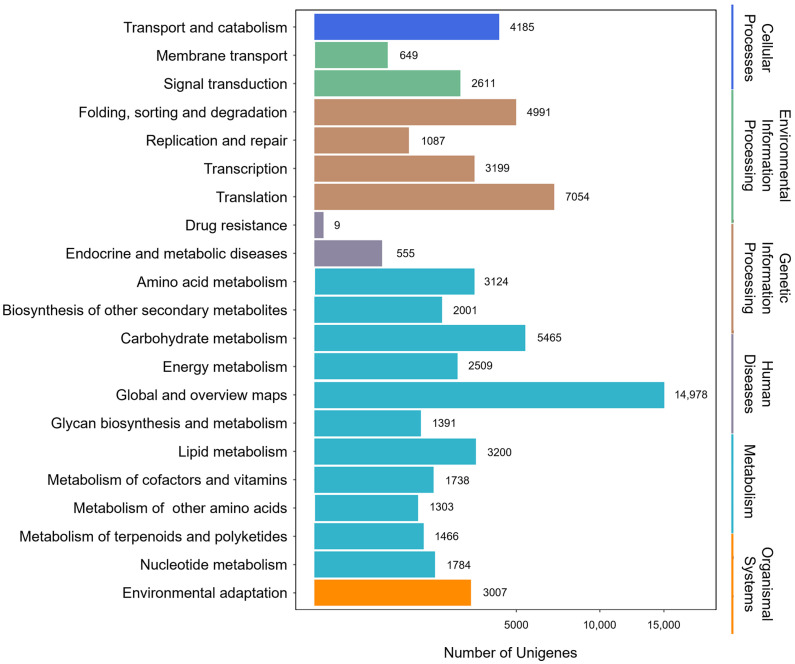
Classification of unigenes based on the KEGG functional pathways.

A total of 66,246 sequences were assigned to 21 KEGG pathways (Figure 2). The metabolism pathway had the highest representation of sequences, followed by genetic information processing, cellular process, environmental information processing, organismal system, and human diseases. Specifically, 38,959 sequences were categorized into the metabolic pathways, e.g., global and overview maps (14,978), carbohydrate metabolism (5465), lipid metabolism (3200), and amino acid metabolism (3124). Moreover, 1466 sequences were assigned to terpenoid and polyketide metabolism. These annotations furnished valuable insights into investigating the specific functions and terpenoid biosynthesis in *P. japonicus*.

### 2.4. Differential Expression of Transcripts in the Three Tissues of P. japonicus

**Figure 3:**
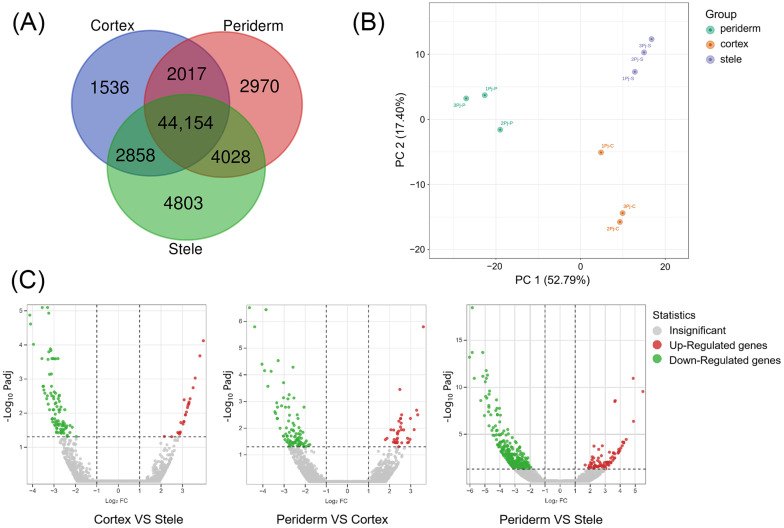
Analysis of genes expressed in three tissues of *P. japonicus.* (**A**) Venn diagram of DEGs. (**B**) PCA of DEGs. (**C**) Volcano plot of DEGs.

In order to better understand the gene expression in the three tissues of *P. japonicus* and their relationships with biological processes, we compared the DEGs among different tissues and analyzed the relationships between DEGs in each tissue. Among 62,366 expressed unigenes, 55,845 unigenes were expressed in the stele, 53,169 in the periderm, and 50,560 in the cortex. A lot of unigenes were identified in more than one tissue. There were 9309 unigenes that were solely expressed in one of the three tissues, 4803 of which were solely expressed in the stele, followed by 2970 in the periderm and 1536 in the cortex. A total of 44,154 (70.8%) unigenes were shared by the three tissues (Figure 3A). 

According to the criteria of a fold change (FC) ≥ 2 and a significant false discovery rate (FDR) ≤ 0.05, 658 DEGs were obtained. According to the results of principal component analysis (PCA), PC 1 explained 52.79% of the variance, while PC 2 accounted for 17.40% (Figure 3B). Overall, the three tissues were separated. Compared with the cortex, there were 25 DEGs showing an up-regulation trend and 92 DEGs showing a down-regulation trend in the periderm. Compared with the periderm, 36 and 81 DEGs showed up-regulation and 96 and 328 DEGs showed down-regulation in the cortex and stele, respectively (Figure 3C). The notion that the transcripts of the three tissues were expressed at different levels in *P. japonicus* was supported by the transcriptional profiles; the transcriptional profile of the stele was evidently different compared with those of the periderm and cortex. These findings suggest that the specificity of metabolite contents may be related to the specificity of gene expression.

### 2.5. Analysis of Genes Implicated in Triterpenoid Saponin Biosynthesis in P. japonicus

**Figure 4:**
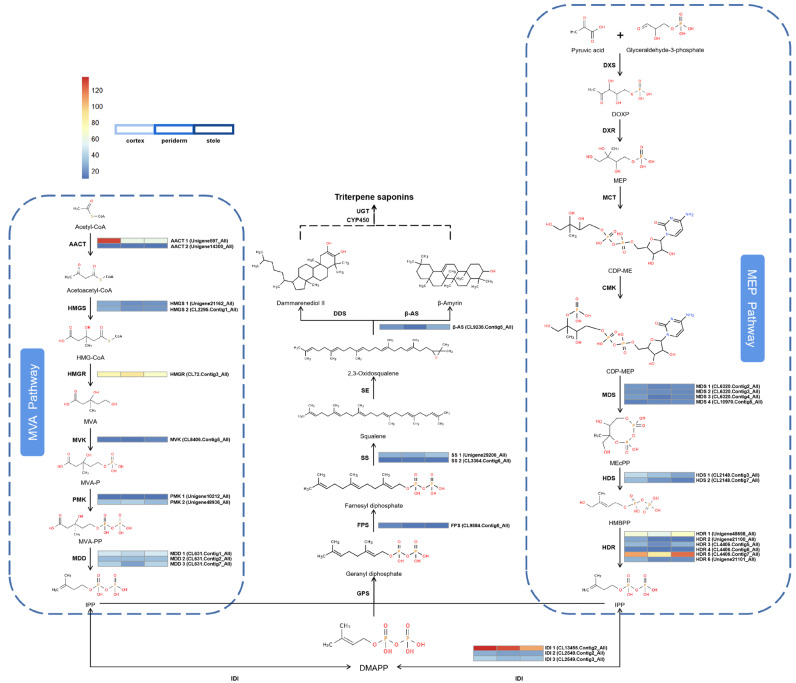
Expression of genes involved in the MVA and MEP pathways for saponin biosynthesis in *P. japonicus*.

To enhance comprehension regarding the regulatory networks involving the biosynthesis, accumulation, and transportation of triterpenoid saponins across various tissues, we focused on genes related to triterpenoid saponin biosynthesis and modification. Analysis of the transcriptome (FPKM ≥ 10) identified 15 genes comprising 119 transcripts that could participate in the biosynthesis of triterpenoid saponin via the MVA and MEP pathways (Figure 4). Furthermore, we identified potential key downstream genes that are responsible for cyclizing and hydroxylating diverse ginsenoside precursors. These candidate genes encode one FPS, two SSs, and one β-AS.

We analyzed the upstream gene expressions in triterpenoid saponin biosynthesis. In the periderm, cortex, and stele; 2 (6.7%), 18 (60.0%), and 10 (33.3%) genes exhibited the highest expression levels, respectively. In order to reveal the potential biological functions and explore the interplay among these genes, a heatmap analysis was performed (Figure 4). The results showed that 18 genes exhibit similar low expression levels across all three tissues, among which five transcripts (AACT 2; HMGS 1, 2; PMK 1; MVK) are involved in the MVA pathway, while nine transcripts (MDS 1, 2, 3, 4; HDS 2; HDR 2, 3, 4, 6) belong to the MEP pathway and four transcripts (IDI 2; SS 2; FPS; β-AS) from the subsequent pathway. Four transcripts (MDD 1, 2, 3; PMK 2) from the MVA pathway and HDS 1 from the MEP pathway, along with IDI 3 and SS 1 from the subsequent pathway, had moderate expression levels in all three tissues, with the majority being expressed at higher levels in the stele than in the periderm or cortex. Five transcripts had relatively high expression levels. Among them, AACT 1 and IDI 1 had the highest expression levels in the cortex, while HDR 1 and HDR 5 were expressed at higher levels in the stele. Additionally, HMGR had the highest expression level in the periderm. In plants, the cortex has metabolic and transport functions while the stele has storage and transport functions [13]. Therefore, higher expression levels of upstream DEGs in the biosynthesis pathway may contribute to the biosynthesis and transport of triterpenoid precursor substances in plants.

### 2.6. Downstream Genes Associated with Saponin Classification in P. japonicus

**Figure 5:**
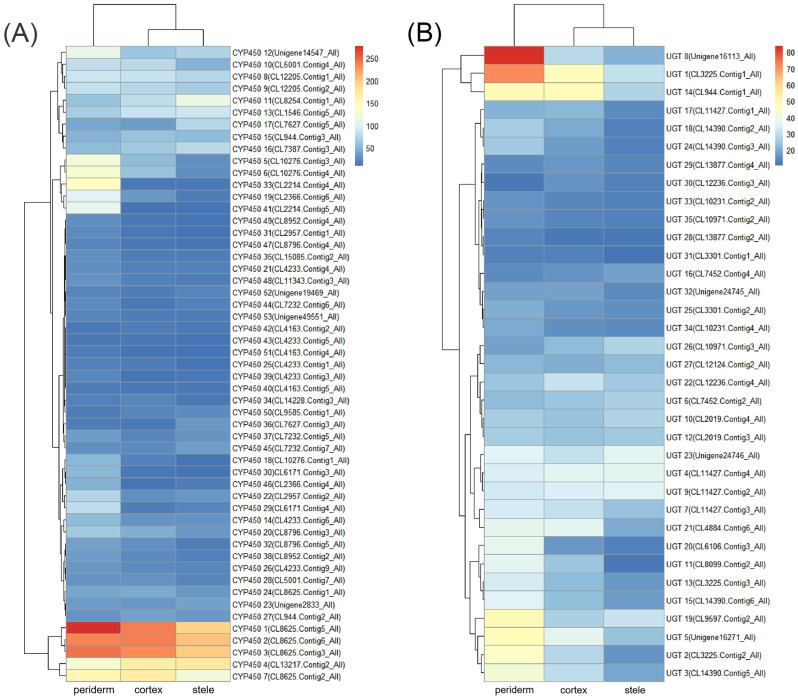
Gene expression of CYPs and UGTs related to saponin biosynthesis in *P. japonicus*. (**A**) Heatmap of CYP gene expression patterns in the three tissues. (**B**) Heatmap of UGT gene expression patterns in the three tissues.

In triterpenoid saponin biosynthesis, the processes of CYPs and UGTs catalyzing the decoration of skeletons are regarded as the rate-limiting steps [14]. In our samples, the expressions of 53 CYP transcripts were significantly different (FPKM ≥ 10) among three tissues, with 37 (69.8%), 7 (13.2%), and 9 (17.0%) transcripts exhibiting maximum expression in the periderm, cortex, and stele, respectively (Figure 5A and Table S4). Additionally, the expressions of 35 UGT transcripts were significantly different, with 21 (60.0%) showing maximum expression in the periderm, 8 (22.9%) in the cortex, and 6 (17.1%) in the stele (Figure 5B and Table S5). These findings indicate the intricate regulation of CYP and UGT gene expression in a tissue-specific manner, potentially affecting the variance in saponin contents across different tissues. The differential expression of CYPs and UGTs suggested that the precursor substances of saponin biosynthesis may undergo further reactions and processing predominantly in the periderm, and the end products might ultimately accumulate in the stele.

### 2.7. Correlation between Saponin Content and Gene Expression

**Figure 6:**
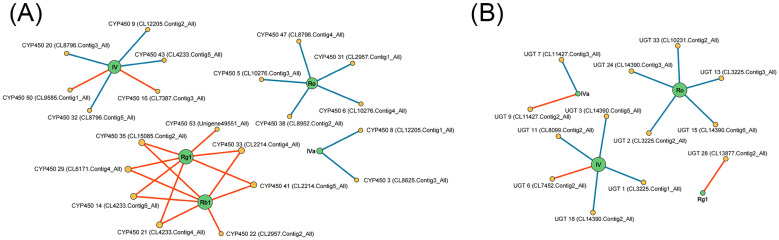
Network diagram of Pearson correlation between gene expression patterns and saponin contents in *P. japonicus* (|R| > 0.80 and *p* < 0.05). Lines colored in red and blue represent positive and negative correlations, respectively. (**A**) CYP gene expression patterns vs. saponin contents. (**B**) UGT gene expression patterns vs. saponin contents.

To explore the correlation between tissue-specific saponin accumulation and the different expression of CYPs and UGTs, Pearson correlation analysis was conducted. The results revealed that 10 CYP genes and 3 UGT genes (FPKM ≥ 10) have significant positive correlations between their expression levels and saponin contents. Among them, 2 CYP genes and 2 UGT genes showed strongly positive correlations with oleanane-type ginsenosides (Ro, IV, and IVa) and 8 CYP genes and 1 UGT gene were strongly positively correlated with dammarane-type ginsenosides (Rg1 and Rb1). Two CYP transcripts (CL7387.Contig3_All and CL9585.Contig1_All) and one UGT transcript (CL7452.Contig2_All) were strongly positively correlated with chikusetsusaponin IV contents (R > 0.80, *p* < 0.05), while one UGT transcript (CL11427.Contig2_All) showed strongly positive correlations with chikusetsusaponin IVa contents (R > 0.80, *p* < 0.05). Additionally, seven CYP transcripts (CL4233.Contig6_All, CL4233.Contig4_All, CL6171.Contig4_All, CL2214.Contig4_All, CL15085.Contig2_All, CL2214.Contig5_All, and Unigene49551_All) and one UGT transcript (CL13877.Contig2_All) were strongly positively correlated with ginsenoside Rg1 contents (R > 0.80, *p* < 0.05). Seven CYP transcripts (CL4233.Contig6_All, CL4233.Contig4_All, CL2957.Contig2_All, CL6171.Contig4_All, CL2214.Contig4_All, CL15085.Contig2_All, and CL2214.Contig5_All) showed strongly positive correlations with ginsenoside Rb1 contents (R > 0.80, *p* < 0.05) (Figure 6, Tables S6 and S7). These results indicate that these genes may be involved in the saponin biosynthesis process and play significant roles, making them promising candidates for further studies to understand the mechanisms of saponin biosynthesis in *P. japonicus*.

### 2.8. Identification of UGT Gene of P. japonicus

**Figure 7:**
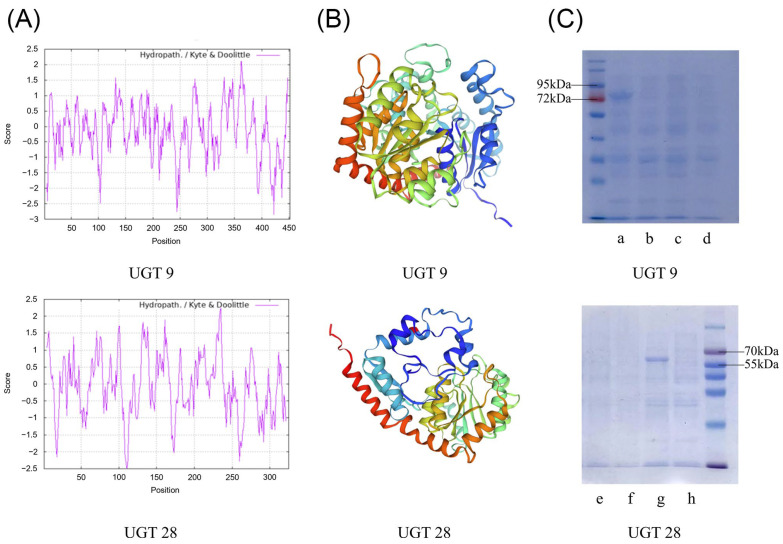
Protein structure prediction and size verification of two recombinant UGT proteins. (**A**) Hydrophilicity analysis of UGT 9 and UGT 28. (**B**) Three-dimensional structure analysis of UGT 9 and UGT 28. (**C**) The SDS-PAGE analysis of the recombinant UGT 9 and UGT 28: (a) 0.5 mM IPTG-induced pET14-UGT 9 expression; (b) control group without induction of pET14-UGT 9 expression; (c) 0.5 mM IPTG-induced pET14-EV expression; (d) control group without induction of pET14-EV expression; (e) same as (c); (f) same as (d); (g), 0.5 mM IPTG-induced pET14-UGT 28 expression; (h), control group without induction of pET14-UGT 28 expression.

Based on the Pearson correlation analysis of *P. japonicus*, we cloned three *UGT* genes that were strongly associated with saponin contents, among which *PjUGT* (*UGT 9*, CL11427.Contig2_All) and *UGT 28* (CL13877.Contig4_All) were successfully cloned (Figure S4). In order to verify the size of the translation product, we cloned and sequenced the *UGT 9* and *UGT 28* (Table S8), whose ORFs were 1359 bp and 978 bp, respectively (Figure S4), encoding 452- and 325-animo-acid polypeptides, respectively. UGT 9 and UGT 28 had theoretical isoelectric points (pIs) of 5.60 and 5.38, respectively. They were hydrophilic proteins (Figure 7A). UGT 9 had 187 α-helices, 23 β-turns, 73 extended strands, and 169 random coils, while UGT 28 had 113 α-helices, 29 β-turns, 53 extended strands, and 130 random coils. Their predicted three-dimensional structures are shown in Figure 7B. We expressed two UGTs in *E. coli* Rosetta-Gami (DE3) and isolated the protein products. In SDS-PAGE, UGT 9 was about 80 kDa (including the 30.2 kDa His-GFP-tag), and UGT 28 was about 65 kDa (including the 30.2 kDa His-GFP-tag) (Figure 7C). The length of the UGT gene in plants is approximately 1000 to 1500 bp. Since the length of *UGT 28* was shorter than 1000 and considering the gene integrity and functional completeness of the encoded protein, we chose *PjUGT* (*UGT 9*) for further analysis and experiments.

**Figure 8:**
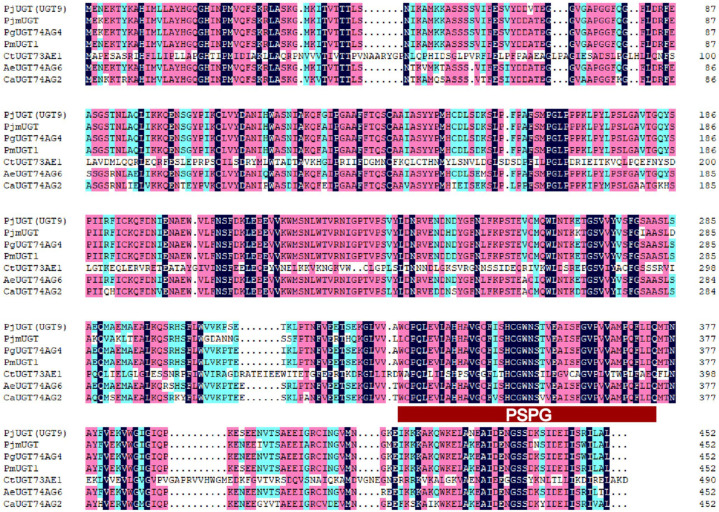
Alignment of deduced amino acid sequences of *PjUGT* (*UGT9*) with other plant *UGTs*: *Panax japonicus* (*PjmUGT*, WME00799), *Panax ginseng* (*PgUGT74AG4*, MH673767), *Panax major* (*PmUGT1*, ON003974), *Carthamus tinctorius* (*CtUGT73AE1*, AJT58578), *Arnebia euchroma* (*AeUGT74AG6*, OK094513), and *Centella asiatica* (*CaUGT74AG2*, MF471461). The UGTs’ signature PSPG motifs are marked with a red rectangle. Fully conserved residues are highlighted in dark blue, strongly conserved (score = 0.75) ones in pink, and weakly conserved (score = 0.5) ones in light blue.

The *UGT* gene exhibits strong conservation in plants, particularly near the stop codon, where there is a highly conserved sequence encoding 44 amino acids known as the Plant Secondary Product Glycosyltransferase (PSPG) motif [15]. Comparisons of amino acid sequences revealed that *PjUGT* (*UGT9*) exhibited 77.94% identity with its homologs in *P. japonicus* (*PjmUGT*), *P. ginseng* (*PgUGT74AG4*), *P. major* (*PmUGT1*), *C. tinctorius* (*CtUGT73AE1*)*, A. elata* (*AeUGT74AG6*), and *C. asiatica* (*CaUGT74AG2*) (Figure 8). Each *UGT* carries the conserved 44-residue PSPG motif at its C-terminus.

**Figure 9:**
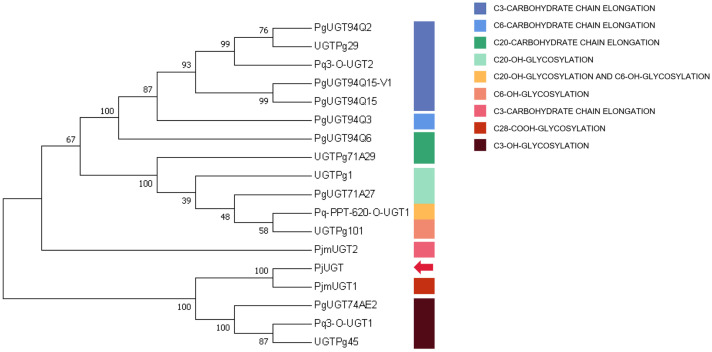
Phylogenetic analysis of *Panax japonicus* UGT. The ClustalW algorithm was utilized for sequence alignment, and the tree was constructed using the neighbor-joining method. The red arrow indicates *PjUGT* (*UGT9*). The accession numbers used are given in Table S2.

Based on the forecasted amino acid sequences of *PjUGT* and other plant *UGTs* that display specificity for C3, C6, C20, and C28 sites of glycosylation, a phylogenetic tree was constructed. *PjUGT* clustered with C28-COOH cluster (Figure 9), and it may be related to C28-COOH glycosylation of triterpene saponins. However, further biochemical experiments are required to precisely determine the function of *PjUGT*.

### 2.9. Functional Characterization of Recombinant PjUGT

**Figure 10:**
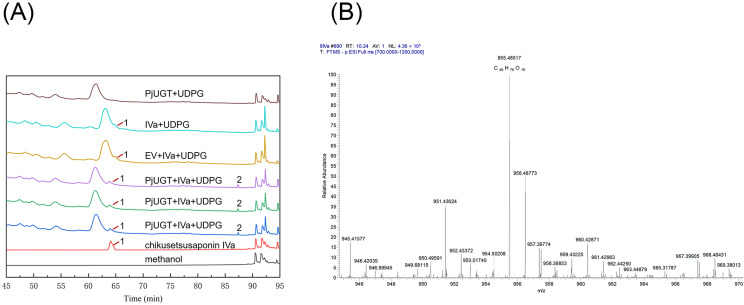
HPLC and LC/MS analysis of the glycosylation activity of recombinant *PjUGT* protein. (**A**) Identification of transformation product by HPLC analysis: (1) peak of chikusetsusaponin Iva and (2) peak of new product. (**B**) Identification of transformation product by LC/MS analysis.

Using chikusetsusaponin IVa as the substrate and UDPG as the glycosyl donor, the enzyme activity assays of the *PjUGT* protein were conducted, and the transformation products were determined by HPLC and LC/MS. Three control groups were also employed: control group 1 included the medium, chikusetsusaponin IVa, UDPG, and yeast transformed with an empty vector (EV); control group 2 consisted of the medium, chikusetsusaponin IVa, and UDPG; and control group 3 included the medium, UDPG, and yeast transformed with *PjUGT* gene. After oscillating fermenting the experimental groups and control groups for three days at 28 °C, a novel product was detected in HPLC (Figure 10A and Figure S5A). Based on the currently known saponin biosynthesis pathway [16], the transformation product may be ginsenoside Ro or a glycosylated intermediate that has not been clearly identified. To clarify the enzymatic reaction product, we performed a comparative analysis with a ginsenoside Ro standard. The HPLC results indicate that the retention time of the new product differed from that of Ro (Figure S5B), suggesting that the product may be a metabolic intermediate in the biosynthesis pathway. To further explore whether the product is a glycosylated compound, LC/MS was employed to further validate the product (Figure 10B). As the molecular formula of chikusetsusaponin IVa is C_42_H_66_O_14_, the glycosylated product could have the molecular formula C_48_H_76_O_19_, resulting in a theoretical molecular weight of 956. Therefore, we searched the peak with a *m*/*z* of 956 in the MS results, and there is a peak with a *m*/*z* of 955.48517 at the retention time of 10.24 min, suggesting a molecular formula of C_48_H_76_O_19_. Compared to chikusetsusaponin IVa, the difference between the two formulas is C_6_H_10_O_5_, which matches the molecular formula of a glucosyl moiety. Therefore, we speculated that *PjUGT* may catalyze the glycosylation of chikusetsusaponin IVa.

## 3. Discussion

### 3.1. Tissue-Specific Expression Patterns of Saponins in P. japonicus

In the rhizomes of plants, different tissues serve distinct functions. The periderm, located at the outermost layer, plays a crucial role in protecting the plant against pathogen invasion and preventing water loss [17]. The cortex is involved in metabolic activities and also contributes to the transport of nutrients towards the stele [13]. And the stele is primarily responsible for the storage and transport of water and nutrients [18]. These distinct functions of the periderm, cortex, and stele may influence the distribution of bioactive compounds, such as saponins, within the tissues. In the *Panax* genus, more than 280 distinct ginsenosides have been isolated and identified [6]. Studies have shown that ginsenosides are unevenly distributed in the different parts and tissues of *Panax* plants. For instance, the periderm of *Panax ginseng* has higher average contents of various ginsenosides compared to the cortex and stele [19]. And in the roots of *P. quinquefolius* and *P. notoginseng*, the saponin distribution was different in the periderm, phloem, and xylem, demonstrating tissue specificity [20]. In this study, we investigated five widely studied saponins with high contents in *P. japonicus* [21] and analyzed their differential expression patterns across different rhizome tissues. We noted that the five saponins had a higher total accumulation in the stele than in other two tissues, which may be related to the stele’s function in storage and transport [18]. Such knowledge enhances our understanding of metabolic regulation in different tissues, offering a foundation for future studies on tissue-specific gene expression and the development of targeted strategies to exploit the medicinal properties of *Panax* plants. Therefore, studies on the different tissues of the rhizomes can help us better exploit the medicinal value of *P. japonicus* rhizomes.

### 3.2. Tissue-Specific Expression Patterns of Gene Expression Related to Saponin in P. japonicus

Genes associated with saponin biosynthesis were extensively identified and displayed various expression patterns across different parts or tissues within the Panax genus [19]. The transcripts associated with the upstream pathway of triterpenoid saponin biosynthesis in *P. quinquefolius* and *P. notoginseng* showed tissue-specific expression patterns. The ranking of the expression levels across the different tissues of *P. quinquefolius* from high to low was periderm, xylem, and phloem, with 21 (46.7%), 19 (42.2%), and 5 (11.1%) transcripts, respectively. Meanwhile, the transcripts in the phloem, xylem, and periderm of *P. notoginseng* exhibited the highest expression levels, comprising 15 (39.5%), 12 (31.6%), and 11 (28.9%) transcripts, respectively [20]. In this study, 119 transcripts with FPKM ≥ 10 were identified in the saponin biosynthesis of *P. japonicus*. In the upstream pathway of triterpenoid saponin biosynthesis in the periderm, cortex, and stele of *P. japonicus*, 2 (6.7%), 18 (60.0%), and 10 (33.3%) transcripts were most highly expressed (FPKM ≥ 10). These results suggest that genes associated with saponin biosynthesis have tissue-specific expression patterns in the rhizomes of *P. japonicus*.

The main downstream enzymes regulating triterpenoid saponin biosynthesis are CYPs and UGTs. Their differential expressions may be related to the tissue specificity of saponin content. CYP introduces oxygen atoms from oxygen molecules to substrates, which involves the biosynthesis of a variety of specialized metabolites in plants [22]. For example, CYP716A53v2 can catalyze hydroxylation at the C-6 position of protopanaxadiol in dammarane-type saponin biosynthesis, leading to the formation of protopanaxatriol [22,23]. Saponin glycosylation catalyzed by UGTs is generally regarded as the last step in triterpenoid saponin biosynthesis, which is essential for the diversity and bioactivity formation of triterpenoid saponins [24]. Four UGTs specifically recognized UDP-glucuronosyl in *P. zingiberensis* and catalyzed the glucuronidation at the C3 site of oleanolic acid [25]. As for the periderm, cortex, and stele in this study, 37 (69.8%), 7 (13.2%), and 9 (17.0%) CYP transcripts had the highest expressing levels, respectively, while 21 (60.0%), 8 (22.9%), and 6 (17.1%) UGT transcripts were maximally expressed, respectively. In plants, secondary metabolites often play a defensive role against pests and animals [19]. In this study, the majority of CYP and UGT transcripts showed the highest expression levels in the periderm. As the periderm has the function of protection, the higher expression levels of these genes may help the further synthesis and processing of saponins in periderm, potentially improving the protectivity of the periderm. According to these results, CYPs and UGTs had tissue-specific expression patterns in *P. japonicus* that are crucial for the structural diversity and tissue-specific distribution of triterpenoid saponins in *Panax* plants.

### 3.3. Co-Expression Analysis of Genes Related to Saponin Accumulation

The differential expression of key biosynthetic genes may be associated with variations in tissue-specific saponins in *P. japonicus*. Pearson correlation analysis was conducted to investigate the associations between the high expression genes (FPKM ≥ 10) and saponin accumulation in *P. japonicus* (Figure 6). In *P. notoginseng*, the CYP716A47 gene had been identified and found to be significantly correlated with the ginsenosides Rc and Rb2 [26]. Furthermore, RNA interference targeting the CYP716A47 gene in *P. ginseng* and *P. quinquefolius* hairy roots resulted in reduced ginsenoside synthesis [27], which confirms the significant correlation between the gene and saponins. In this study, the expression levels of 10 CYP genes and 3 UGT genes (FPKM ≥ 10) were found to be significantly positively correlated with saponin contents. These genes may encode enzymes that are involved in saponin biosynthesis, and further investigation is required to comprehensively elucidate the biological functions of these genes. These findings also provide some references for the in vitro synthesis of triterpenoid saponins as well as the trait improvement of *P. japonicus*.

### 3.4. Functional Regulation of the Triterpenoid Saponin Biosynthesis Pathway and Key Enzyme Genes

Almost all genera of flowering plants synthesize saponins [28]. Currently, approximately 150 naturally occurring ginsenosides have been identified in *Panax* species [29], each of which differs in the number, position of linkage, and type of sugar moieties thereof [30]. The physicochemical properties and biological activities of triterpenes in plants can be modified by glycosylation [31], which is catalyzed by a superfamily of UGT, representing a crucial mechanism for regulating the bioactivity and storage of phytochemicals in plants [24,32]. It is worth noting that several genes related to the biosynthesis of ginsenoside were annotated as *UGTs*, among which three UGTs were found to strongly correlate with the content of saponins through Pearson analysis. We successfully cloned and sequenced *PjUGT* (*UGT 9*, CL11427.Contig2_All) and *UGT 28* (CL13877.Contig4_All) genes. However, we were unable to clone *UGT 6* (CL7452.Contig2_All) due to the inability to amplify a correctly sized product from the cDNA. Possible reasons for this include poor primer specificity, quality issues with the cDNA template, or other template-related issues. We confirmed the molecular weight of the recombinant proteins to be approximately 50 kDa and 35 kDa, respectively, through the induction of *PjUGT* and *UGT 28* gene expression in *E. coli*. The sequence alignment and phylogenetic analysis suggest that the function of *PjUGT* was related to glycosylation at the C28-COOH site of saponins. 

In order to explore the catalytic activity of *PjUGT*, we transformed a recombinant plasmid containing the *PjUGT* gene into yeast and conducted fermentation experiments. In this study, control groups were set up to exclude the following possibilities: the product was produced by the yeast itself, the product was generated from substrate decomposition, or the product was formed by *PjUGT* catalyzing other components in the medium. The results confirmed that *PjUGT* had the ability to catalyze chikusetsusaponin IVa and produce a new product.

The biosynthesis of triterpenoid saponins involves the modification and processing of many enzymes, and the pathway is still incomplete in current research. Research indicates that ginsenoside Ro is the currently known product generated from the glycosylation of chikusetsusaponin IVa [16]. The HPLC results showed that the new product catalyzed by *PjUGT* differs from the known product ginsenoside Ro, suggesting that it may be an unidentified metabolic intermediate. Our LC/MS results showed that there exists a product with the molecular formula C_48_H_76_O_19_, which corresponds to the molecular formula of the product resulting from the glycosylation of chikusetsusaponin IVa. Therefore, we speculate that *PjUGT* could catalyze the glycosylation of chikusetsusaponin Iva; however, further studies are needed to explore the catalytic site of *PjUGT*.

## 4. Materials and Methods

### 4.1. Plant Materials and Reagents

Three-year-old *P. japonicus* plants were obtained in August from Enshi, Hubei Province, China; they were authenticated as *P. japonicus* by Professor Dingrong Wan of South-Central University for Nationalities in China. The plant rhizomes were cleaned and sorted into three tissues, periderm, cortex, and stele, with the samples in triplicate for each tissue type, then each sample was stored at −80 °C until used.

### 4.2. Targeted Metabolites Analysis

#### 4.2.1. Sample Solution Preparation

Three samples were prepared for each tissue. Each sample of 0.5 g was weighed and then pulverized into powder. After powder drying to a constant weight, 25 mL of methanol was added for each 200 mg of sample. The mixture was soaked for 2 h. After ultrasonic extraction for 45 min, the sample was thoroughly mixed and 0.22 μm pore size membrane filters were used to filter them. Afterward, the samples were analyzed using HPLC following the procedure outlined below.

#### 4.2.2. Standard Preparation

The standards (ginsenoside Ro, chikusetsusaponin IVa, chikusetsusaponin IV, ginsenoside Rg1, and ginsenoside Rb1) were weighed and dissolved in methanol to a concentration of 0.1 mg/mL, and then stored at 4 °C.

#### 4.2.3. HPLC Conditions

A gradient elution approach was conducted for HPLC using an Agilent 1260 Infinity II high-performance liquid chromatography system with a Hypersil BDS C18 column (4.6 mm × 250 mm, 5 μm, Thermo Fisher Scientific, Waltham, MA, USA). The mobile phase comprised acetonitrile (A) and 0.05% phosphoric acid aqueous solution (B). The gradient settings were as follows: 0–5 min, 10% A; 5–10 min, 10% → 15% A; 10–15 min, 15% → 20% A; 15–28 min, 20% → 25% A; 28–33 min, 25% → 30% A; 33–68 min, 30% → 35% A; 68–81 min, 35% → 50% A; 81–91 min, 50% → 90% A; 91–96 min, 90% A; 96–105 min, 90% → 10% A; 105–115 min, 10% A. The column temperature was 30 °C, and the flow rate was maintained at a constant 1.0 mL/min. The injection volume of samples was 10 μL, and the wavelength used for detection was 203 nm.

### 4.3. Transcriptomic Analysis

#### 4.3.1. cDNA Synthesis and Illumina Sequencing

The samples used for transcriptomic analysis were those described in Section 4.1., with samples in triplicate for each tissue type. The total RNA was extracted from nine samples using Trizol, Thermo Fisher Scientific, Waltham, MA, USA. The methodology followed was similar to that of Rio [33], with slight modifications. Approximately 50–100 mg of the sample was ground in liquid nitrogen and subsequently mixed with 1 mL of Trizol reagent. After precipitation with isopropanol, the resulting precipitate was washed twice with 1 mL of 75% ethanol. Once the RNA precipitate was dried, it was resuspended in 30 μL of RNase-free water and stored at −80 °C. The raw data were obtained through Illumina sequencing and CASAVA base recognition. Clean reads were generated by removing vector contaminated reads, empty reads, and unknown sequence reads. The clean reads were assembled using Trinity [34], and the unigenes were obtained by using Tgicl to cluster and remove redundancy [35].

#### 4.3.2. Gene Annotation and Identification of DEGs

All full-length transcripts identified using SMRT sequencing were aligned using BLAST software (version 2.2.26) [36] against the NCBI Nr, SwissProt, GO, COG, KOG, Pfam, and KEGG databases, respectively, to obtain the most comprehensive annotation information. The unigenes were annotated using the alignment results with the lowest E-value. 

The FPKM technique was used to quantify the gene expression levels in order to discover DEGs between different samples. DESeq (1.10.1) was utilized for gene differential expression analysis, employing read counts derived from the gene expression level analysis. The *p* < 0.05 level was commonly used as the benchmark for differential gene selection.

#### 4.3.3. Pearson Analysis and Visualization of Gene Co-Expression

The correlation between saponin content and gene expression can be obtained by calculating the Pearson correlation coefficient, which can reflect the closeness of the correlation between two variables; the calculation formula is as follows:

$$r=\frac{\sum_{i=1}^{n}(x_{i}-\bar{x})(y_{i}-\bar{y})}{\sqrt{\sum_{i=1}^{n}{\left(x_{i}-\bar{x}\right)}^{2}\sum_{i=1}^{n}{\left(y_{i}-\bar{y}\right)}^{2}}}$$

In the equation, *n* is the number of samples and *x_i_* and *y_i_* denote the expression levels of the variables *x* and *y* at the *i*-th sample point, respectively. When *r* is equal to 0, there is no linear relationship between the two variables. When 0 < *r* < 1, there is a certain linear relationship between the two variables. When *r* = 1, the correlation is completely positive, while *r* = −1 means a negative correlation. 

The calculation of the Pearson correlation coefficient and the drawing of the correlation network diagram were conducted using the Metware Cloud (https://cloud.metware.cn, accessed on 24 June 2024).

### 4.4. PjUGT Gene Cloning

#### 4.4.1. RNA Extraction and cDNA Synthesis

Trizol was used to isolate total RNA from *P. japonicus* samples [33], and the specific methods were described above. RNase-free agarose gel electrophoresis and an ultraviolet spectrophotometer (OD_260_/OD_280_ of 1.8–2.2) were used to confirm the quality of RNA. The RNA was then reverse transcribed into cDNA.

#### 4.4.2. Cloning of UGT Genes

According to the transcriptome data, specific primers were designed, and the sequences are listed in Table S1. The cDNA templates were used to amplify the ORFs of genes using KOD -Plus- Neo (TOYOBO, Shanghai, China). The amplified fragments were verified using agarose gel electrophoresis and then recovered according to the instructions of the DNA Gel Extraction & DNA Purification Kit (Simgen, Hangzhou, China). 

Recombinant plasmids were constructed via blunt-end ligation and then transformed into *E. coli* DH5α cells. For sequencing, only positive colonies were chosen.

ProtScale (https://web.expasy.org/protscale/, accessed on 2 July 2024) was used for the hydrophobicity analysis of recombinant UGT proteins. The secondary structure of the proteins was predicted by using SOPMA (https://npsa-prabi.ibcp.fr/cgi-bin/npsa_automat.pl?page=/NPSA/npsa_sopma.html, accessed on 2 July 2024). Homology modeling of the proteins was conducted using SWISS-MODEL (https://swissmodel.expasy.org/, accessed on 2 July 2024).

To predict their function, the gene sequences were aligned using BLAST against publicly available nucleotide and protein databases (www.ncbi.nlm.nih.gov/BLAST, accessed on 4 July 2024). DNAMAN v9 software (LynnonBiosoft, Quebec, Canada) was employed for multiple sequence alignments, and the *UGT* genes subjected to multiple sequence alignment with *PjUGT* are listed in Table S2. Phylogenetic analysis was conducted with the ClustalW tool from the MEGA7 package (www.megasoftware.net/, accessed on 4 July 2024). The credibility of tree branches was assessed by a bootstrap test of 1000 replicates [37]. The UGT genes used for phylogeny are listed in Table S2.

### 4.5. Prokaryotic Expression

To construct an expression plasmid vector for *E. coli*, *UGT* genes were amplified and cloned into pET14b by homologous recombination. The primer pairs used were designed and are given in Table S1. The recombinant plasmids were transformed into *E. coli* Rosetta-Gami (DE3) cells. The recombinant protein was induced to express at 16 °C for 16 h using 0.5 mM IPTG. After centrifuging the cells to extract their contents and resuspending them in PBS solution, the size of the recombinant protein was verified using SDS-PAGE and Coomassie Brilliant Blue staining.

### 4.6. Enzyme Activity Analysis of Recombinant PjUGT

The pESC-Ura-*PjUGT* recombinant plasmid was constructed by homologous recombination. The primer pairs used are given in Table S1. The pESC-Ura-*PjUGT* vectors were transformed into yeast BY4741 cells via the electroporation method. The single clone was inoculated into 5 mL SD-URA medium and cultivated at 28 °C for 24 h. The yeast was then collected and added to 25 mL of special YPD medium (1% yeast extract, 2% peptone, and 2% galactose) and cultivated at 28 °C for 48 h. After that, 0.1 mM chikusetsusaponin IVa and 0.1 mM UDPG were added and cultivated at 28 °C for three days. The fermentation broth was then combined with an equal volume of methanol and given a 20 min ultrasonication for product extraction. Finally, the supernatant, obtained after 10 min of centrifugation at 12,000 rpm, was subjected to HPLC analysis.

### 4.7. Identification of UGT Transforming Products by HPLC and LC–MS

The products were detected and identified using the aforementioned HPLC methods. In addition, the product was detected in negative ionization mode using UPLC-Q Exactive liquid chromatography mass spectrometry (LC/MS). A HypersII BDS C18 column (250 mm × 4.6 mm, 5 μm) was used. The chromatographic mobile phase containing acetonitrile (A) and 0.1% formic acid (D) was applied as follows: 0–10 min, 10% A; 10–40 min, 50% A; 40–60 min, 90% A. The column temperature was 30 °C, and the flow rate was maintained at a constant 0.8 mL/min. The injection volume of the samples was 10 μL. 

As for the mass spectrometry conditions, the data were collected in negative ion mode for full scan analysis. The sheath gas flow rate was set to 40.00 arb, while the aux gas flow rate was 20.00 arb and the sweep flow rate was maintained at 0.00 arb. The spray voltage was 3.81 kV, with a spray current of 3.80 μA. The capillary temperature was kept at 325.0 °C, and the aux gas heater temperature was 350.0 °C. The MS acquisition range was from 700 to 1200.

### 4.8. Statistical Analysis

Student’s *t*-test was conducted to identify significant differences. Genes with |log2(FoldChange)| > 1 and p-value < 0.001 were selected as DEGs. PCA and volcano plot analysis were performed using the Metware Cloud (https://cloud.metware.cn, accessed on 20 June 2024). R version 4.4.0 (www.r-project.org, accessed on 18 May 2024) was used for creating heatmaps. A threshold of *p* < 0.05 was applied to determine statistical significance.

## 5. Conclusions

In summary, the distribution and contents of triterpenoid saponin exhibited tissue-specific patterns in *P. japonicus* rhizomes. Metabolomic and transcriptomic analyses identified saponin biosynthesis genes in the periderm, cortex, and stele of *P. japonicus* rhizomes. The Pearson correlation analysis provided clues regarding the influence of CYP and UGT transcripts on different types of saponins in the rhizomes. One potential candidate gene, *PjUGT*, was identified for further investigation regarding its functions in triterpenoid saponin biosynthesis; its function in saponin biotransformation was preliminarily explored. Together, these findings help us to understand the mechanism of triterpenoid saponin biosynthesis in different rhizome tissues of *P. japonicus* at the molecular level and serve as a valuable reference for the further exploration and utilization of the rhizome resources of *P. japonicus*.

## Data Availability

The data presented in this study are available in the article and Supplementary Materials.

## Supplementary Materials

The following supporting information can be downloaded at: https://www.mdpi.com/article/10.3390/molecules29204936/s1. Figure S1: Summary of genes annotated to NR, KEGG, COG, and SwissProt; Figure S2: Summary of Clusters of Orthologous Groups of proteins (COG) annotation for all Unigenes; Figure S3: Summary of Gene Ontology (GO) annotation for all Unigenes; Figure S4: 1% agarose gel electrophoresis results; Figure S5: HPLC chromatograms of enzymatic reaction products and comparison with ginsenoside Ro standard; Table S1: The gene primer sequences used in the article; Table S2: UGT genes used for multiple sequence alignment and phylogeny; Table S3: Summary of the transcripts and the assembly results for *P. japonicus*; Table S4: Expression of 54 CYP450 transcripts in periderm, cortex, and stele; Table S5: Expression of 35 UGT transcripts in periderm, cortex, and stele; Table S6: R value of the correlation analysis between the expression of CYP450 and UGT transcripts and saponins contents; Table S7: *p* value of the correlation analysis between the expression of CYP450 and UGT transcripts and saponin contents; Table S8: The gene sequences after sequencing.
