# Supplementary material for: Chemical and Transcriptomic Analyses Provide New Insights into Key Genes for Ginsenoside Biosynthesis in the Rhizome of Panax japonicus C. A. Meyer
Source: Molecules. 2024 Oct 18;29(20):4936. doi: 10.3390/molecules29204936 (PMC11510602; doi:10.3390/molecules29204936)
Supplement: Supplementary file 1 [file molecules-29-04936-s001.zip › Figure S1-S5.pdf]

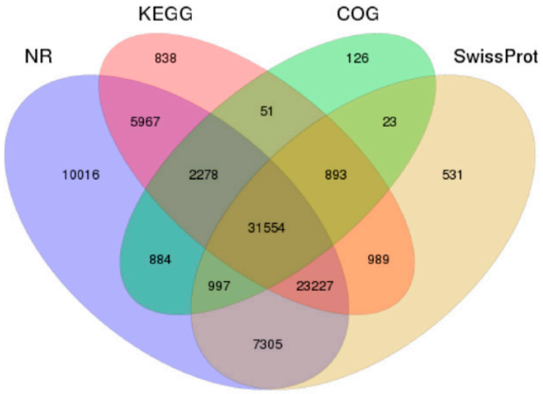

Figure S1: Summary of genes annotated to NR, KEGG, COG and SwissProt

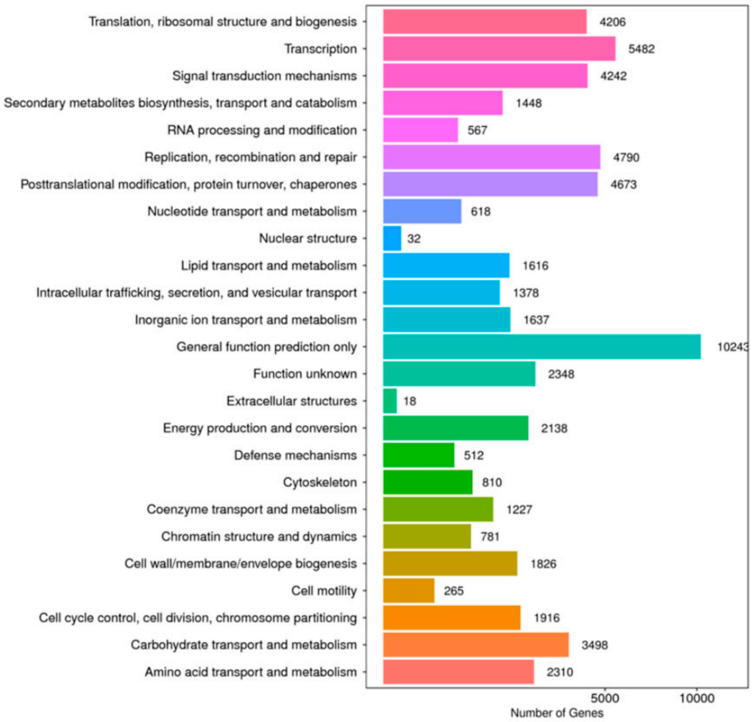

Figure S2: Summary of Clusters of Orthologous Groups of proteins (COG) annotation for all Unigenes

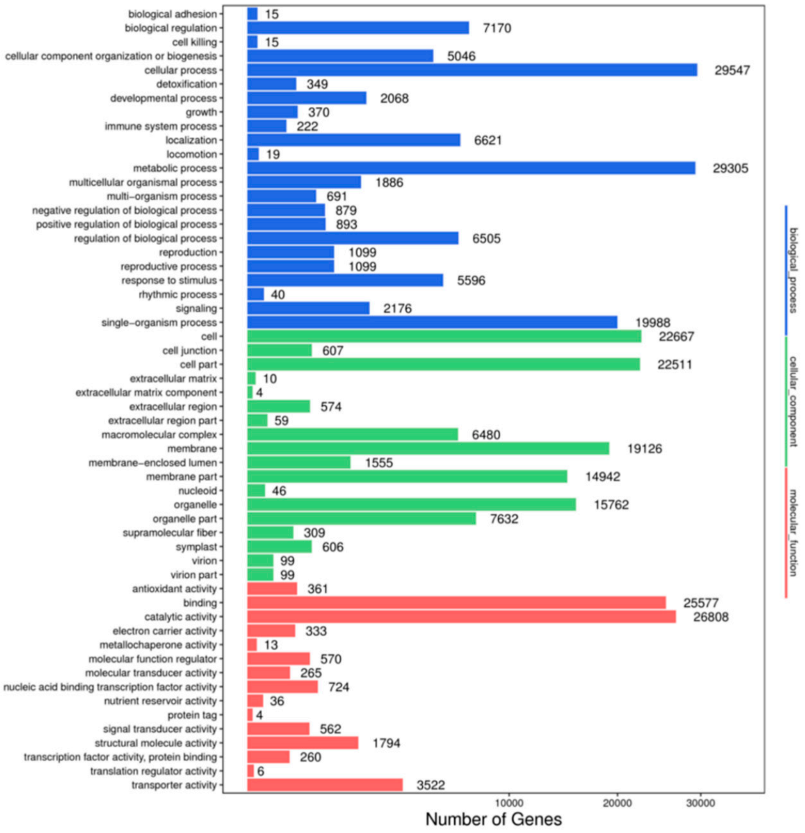

Figure S3: Summary of Gene Ontology (GO) annotation for all Unigenes

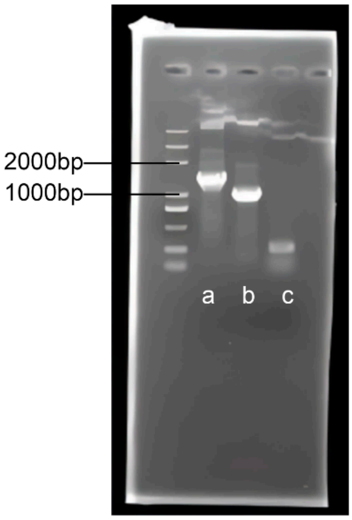

Figure S4: 1% agarose gel electrophoresis results. (a) UGT9; (b) UGT28; (c) UGT6

(A)

(B)

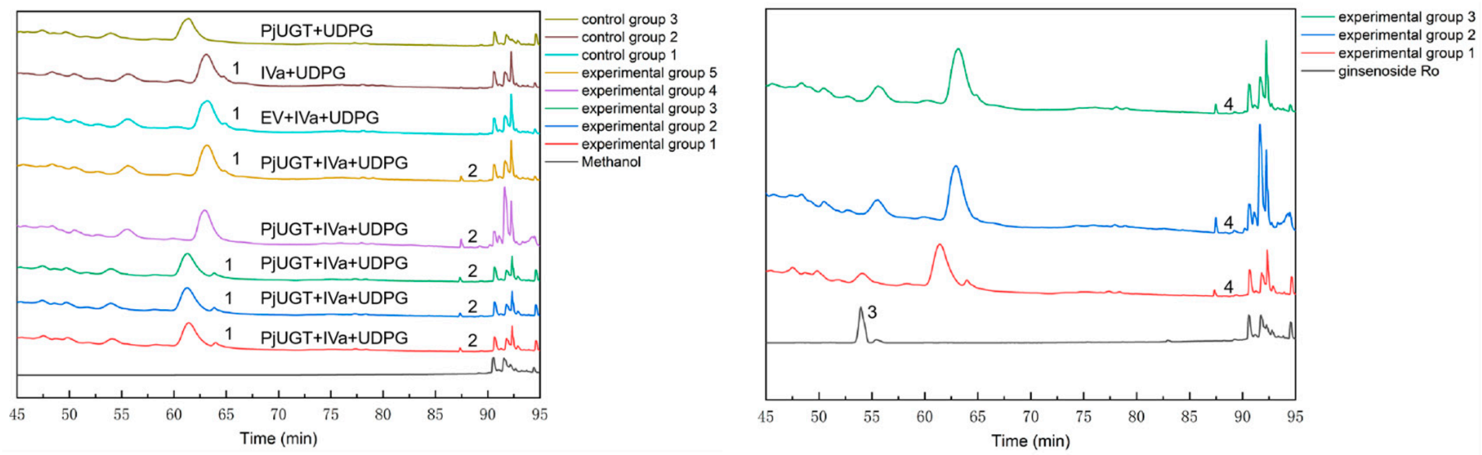

Figure S5: HPLC chromatograms of enzymatic reaction products and comparison with ginsenoside Ro standard. (A) HPLC analysis of the products from five parallel enzymatic activity assays of recombinant *PjUGT*: (1) peak of chikusetsusaponin IVa; (2) peak of new product. (B) Comparative HPLC chromatogram of the enzymatic reaction product and the ginsenoside Ro standard: (3) peak of ginsenoside Ro; (4) peak of new product.
