# Supplementary material for: Chemical and Transcriptomic Analyses Provide New Insights into Key Genes for Ginsenoside Biosynthesis in the Rhizome of Panax japonicus C. A. Meyer
Source: Molecules. 2024 Oct 18;29(20):4936. doi: 10.3390/molecules29204936 (PMC11510602; doi:10.3390/molecules29204936)
Supplement: Supplementary file 1 [file molecules-29-04936-s001.zip › Table S1.pdf]

Table S1. The gene primer sequences used in the article

Primer name	Primer sequence (5' to 3')
UGT9-F	ATGGAGAATGAGAAAACTTATAAAG
UGT9-R	TTAGAGTGCCAGAATCCGA
UGT28-F	ATGGCTTTTCTTGGGCCGC
UGT28-R	TTAACTCATCAATTGGGATTTCCTCAT
pET14-*UGT9*-F	TGTACTTCCAGGGTCATATGATGGAGAATGAGAAA ACTTATAAAGCTCATATCAT
pET14-*UGT9*-R	CTTTGTTAGCAGCCGGATCCTTAGAGTGCCAGAATC CGAGAAATAATTTCATCA
pET14-*UGT28*-F	TGTACTTCCAGGGTCATATGATGGCTTTTCTTGGGC CG
pET14-*UGT28*-R	CTTTGTTAGCAGCCGGATCCTTAACTCATCAATTGG GATTTCCTCATTTTTACAAGG
pESC-*PjUGT*-F	TACGACTCACTATAGGGCCCATGGAGAATGAGAAA ACTTATAAAGCTCATATCATGC
pESC-*PjUGT*-R	ATCTTAGCTAGCCGCGGTACTTAGAGTGCCAGAAT CCGAGAAATAATTTCATCA
