# Supplementary material for: Chemical and Transcriptomic Analyses Provide New Insights into Key Genes for Ginsenoside Biosynthesis in the Rhizome of Panax japonicus C. A. Meyer
Source: Molecules. 2024 Oct 18;29(20):4936. doi: 10.3390/molecules29204936 (PMC11510602; doi:10.3390/molecules29204936)
Supplement: Supplementary file 1 [file molecules-29-04936-s001.zip › Table S3.pdf]

Table S3. Summary of the transcripts and the assembly results for *P. japonicus*

Item	No. of Sequences
Clean reads (M)	366.6572
Clean bases (G)	54.9985
No. of contig＞500bp	777,642
Total unigenes	119,882
Total length (bp)	126,146,371
Average contig size (bp)	1,052
N50 contig size (bp)	1,691
GC (%)	41.28
