# Supplementary material for: Chemical and Transcriptomic Analyses Provide New Insights into Key Genes for Ginsenoside Biosynthesis in the Rhizome of Panax japonicus C. A. Meyer
Source: Molecules. 2024 Oct 18;29(20):4936. doi: 10.3390/molecules29204936 (PMC11510602; doi:10.3390/molecules29204936)
Supplement: Supplementary file 1 [file molecules-29-04936-s001.zip › Table S8.pdf]

Table S8. The genes sequence after sequencing

***UGT9* sequence information obtained after sequencing**
ATGGAGAATGAGAAAACTTATAAAGCTCATATCATGCTGCTAGCATATCAT<br>GGGCAAGGTCACATAAATCCGATGGTCCAATTCTCTAAACGTCTTGCTTC<br>TAAAGGAATGAAAATCACCGTAACCACCACACTCTCCAATATCAAGGCC<br>ATGAAAAAGGCATCTTCTAGCTCAGTTATATTTGAATCCGTATATGATGAC<br>GTCACTGAAGGTGGAGTGGGAGCACCTGGAGGATTTCAGGGATTTCTTG<br>ACAGGTTTGAAGCTAGCGGCTCAACAAACTTGGCTCAACTCATCAAGAA<br>ACAAGAAAACTCTGGATACCCTATTAAGTGCCTCGTTTATGATGCTAACAT<br>ACATTGGGCTTCAAATATAGCCAAGCAGTTTGGGATACCTGGGGCTGCTT<br>TTTTCACGCAATCATGTGCTGCTATTGCTAGCTACTACCCAATGCATTGTG<br>ATTTATCAGACAAGTCTCTGCCATTCCCTGCTTTTTCCATGCCTGGATTGC<br>CACCGCCTAAGCTTCCATATCTGCCATCACTTGGTGCTGTTACAGGACAG<br>TACTCCCCAATAATCCGTTTCATATGCAAGCAATTCGACAATATAGAGAAT<br>GCAGAGTGGGTCCTTTTCAACTCCTTTGATAAATTAGAAGAAGAGGTGG<br>TGAAGTGGATGTCAAATCTGTGGACAGTGAGGAATATTGGACCGACTGT<br>GCCATCTGTGTACCTGGACAATCGAGTGGAAAATGACAATGATTACGGTT<br>TCAATCTTTTTAAGCCAAGCACTGAGGTTTGCATGCAGTGGCTCAACACT<br>AAAGAGACTGGGTCAGTTGTGTACGTATCGTTTGGTAGTGCTGCTAGTTT<br>GAGTGCAGAACAGATGGCAGAAATGGCCGAGGCCCTTAAACAAAGCAG<br>ACACAGTTTCTTATGGGTGGTGAAACCATCCGAGACCAAGCTCCCAACT<br>AATTTTGTTGAGGAGACATCAGAAAAGGGACTGGTAGTGGCTTGGTGCC<br>CACAGTTGGAGGTGTTAGCCCATCATGCAGTGGGTTGCTTCATATCGCAC<br>TGCGGATGGAATTCTACTGTAGAGGCAATAAGCTTTGGGGTGCCTGTAGT<br>GGCAATGCCACAGTTTCTAGACCAAATGACAAATGCTTATTTTGTGGAAA<br>AAGTTTGGGGAATTGGAATCCAACCAAAGGAAAGCGAAGAAAATGTTAC<br>AAGTGCTGAAGAGATTGGGAGATGCATCAATGGGGTCATGAATGGAAAG<br>GAGATTAAAAAGAAAGCTAAGCAGTGGAAGGAGTTGGCTAACGAGGCA<br>ATAGATGAAAATGGAAGTTCAGATAAGTCTATTGATGAAATTATTTCTCGG<br>ATTCTGGCACTCTAA

UGT28 **sequence information obtained after sequencing**
ATGGCTTTTCTTGGGCCGCCTTCACTGTTGGTGAGTGGAGAGGATGGTAG<br>ATTGAAGGGTGAGGATTTTACAATTGTGCCCAAGTGGATCCCGTTTCAAT<br>CCGATGTCGTTTTTCGCCTCCATGAGGTGATAAAGTATGTTGATGGTTCAG<br>CTGGGAATGAGTCTGGTGCATCTGATTCAGTAAGATTTGGTGCTTCTATTG<br>GTGGGAGTGACTTGGTTTTGTTTAGAACTTGTGTTGAGTTTGAACCGGA<br>ATGGTTCAATCTTGTTTGTCAGCTTTACCGGAAACCGGTTGTTCCGGTGG<br>GAGTTTTGCCACCAGATAATGATGATTTTGGTAATGAAGAGAAATGGGTT<br>TTTATTAAAGAGTGGTTAGACAAACAGATGGTCAATTCTGTGTTATATGTT<br>GCACTAGGAAGTGAGGCGATACTGAGTCGGGATGAACTCAGTGAGTTAG<br>CTCTCGGGTTGGAGCAGTGTGGGTTGCCTTTCTTTTGGGTGCTAAGAATG<br>CCTCCTGGGTCGACTCAGGATGAGTCACAACTTCTACTTCCCGATGGGTT<br>TGTTGAGCGAGTCAAAAGTCGAGGGGTCGTTCACCAGGGGTGGGTCCC<br>GCAGGTTAAGATACTGAGTCACTCGGCCGTTGGGGGTTTCTTGACTCACT<br>GTGGTTGGAACTCGGTTGCTGAGGCACTCGGTTATGGACGAGTTTTGATA<br>CTTTTTCCGGTGATGAATGACCAAGGTTTGAATGCCAGGTTGTTGAAAGG<br>GAAGAAAGTCGGGGTGGAAATTCCGAGGAATGAGAGAGATGGGGCCTT<br>CACGAGTGACTCGGTGGCGGACTCGGTGCGGATTGCTATGGTGAGTCAA<br>GAGGGCGAGTCGGTGAGGGCTAATGCAAGAGAGATGAGAGGATTGTTTG<br>GACATGGAATTAGGAATGAAAATTGCATTGACAATTTTGTTTCTCACCTT<br>GTAAAAATGAGGAAATCCCAATTGATGAGTTAA
